# Addiction Consult Services, Mortality, and Acute Care Utilization in Inpatients With Opioid Use Disorder: A Secondary Analysis of a Cluster Randomized Clinical Trial

**DOI:** 10.1001/jamanetworkopen.2025.25222

**Published:** 2025-08-06

**Authors:** Yasna Rostam-Abadi, Scarlett Wang, Carla King, Roopa Kalyanaraman Marcello, Gretchen Van Wye, Ellenie Tuazon, Joseph Kennedy, Caroline Cooke, Medha Mazumdar, Thaddeus Tarpey, John Billings, Noa Appleton, Jasmine Fernando, Adetayo Fawole, Samira Siddiqui, Charles Barron, Daniel Schatz, Jennifer McNeely

**Affiliations:** 1 Department of Population Health, NYU Grossman School of Medicine, New York, New York; 2 Department of Psychiatry, Yale School of Medicine, New Haven, Connecticut; 3 NYU Robert F. Wagner Graduate School of Public Service, New York, New York; 4 Office of Behavioral Health, New York City Health + Hospitals, New York, New York; 5 Office of Population Health, New York City Health + Hospitals, New York, New York; 6 Bureau of Vital Statistics, New York City Department of Health and Mental Hygiene, New York, New York; 7 Bureau of Alcohol and Drug Use Prevention, Care, and Treatment, New York City Department of Health and Mental Hygiene, New York, New York; 8 Enterprise Analytics, Mass General Brigham, Boston, Massachusetts

## Abstract

**Importance:**

With acute care utilization and mortality rates increasing among people with opioid use disorder, hospital addiction consult services can provide an important touchpoint for care, potentially leading to improved outcomes.

**Objective:**

To study the effectiveness of interprofessional hospital addiction consultation services on postdischarge acute care utilization and mortality.

**Design, Setting, and Participants:**

In this pragmatic stepped-wedge cluster randomized implementation and effectiveness (hybrid type 1) clinical trial, 6 New York City public hospitals were randomized to an intervention start date, and outcomes were compared during treatment as usual (TAU) and intervention conditions. Participants included adults with hospitalizations identified in Medicaid claims data between October 2017 and January 2021. Eligible patients had an admission or discharge diagnosis of opioid use disorder or opioid poisoning or adverse effects, were hospitalized at least 1 night in a medical or surgical inpatient unit, and were not receiving medication for opioid use disorder before hospitalization.

**Intervention:**

Hospitals implemented the Consult for Addiction Treatment and Care in Hospitals (CATCH) program, an interprofessional inpatient addiction consult service providing specialty care for substance use disorders, with teams consisting of a medical clinician, social worker or addiction counselor, and peer counselor.

**Main Outcomes and Measures:**

Acute care utilization (hospitalizations and emergency department [ED] visits) and mortality rates (all-cause deaths, overdose deaths, and opioid-involved overdose deaths) 1 year after hospital discharge. Data for the eligible patients were analyzed July 2023 to September 2024.

**Results:**

In total, 1355 eligible admissions were identified (968 [71.4%] men; mean [SD] age, 46.6 [12.4] years). A majority of patients (835 [61.5%]) had at least 1 subsequent hospitalization or ED visit. There were 113 deaths, including 34 overdose deaths (30.1%), of which 28 (82.4%) involved opioids. ED admissions were lower in the intervention period compared with TAU (incidence rate ratio, 0.79 [95% CI, 0.72-0.88]; *P* < .001). There were no statistically significant differences between CATCH and TAU periods in numbers of hospitalizations (incidence rate ratio, 0.99 [95% CI, 0.87-1.13]) or mortality (eg, hazard ratio for all-cause death, 1.14 [95% CI, 0.98-1.92]).

**Conclusions and Relevance:**

In this prespecified secondary analysis of a cluster randomized clinical trial, postdischarge ED visits decreased with the CATCH program, highlighting the potential of hospital-based addiction consult services to address needs of patients with opioid use. Nonetheless, high rates of acute care utilization and mortality persisted, underscoring the need for comprehensive care strategies that extend beyond the hospital walls, and addressing the complex health and social needs of individuals with opioid use.

**Trial Registration:**

ClinicalTrials.gov Identifier: NCT03611335

## Key Points

**Question:**

Do hospital addiction consult services decrease 1-year acute care utilization or mortality among patients with opioid use disorder?

**Findings:**

This secondary analysis of a cluster randomized clinical trial with 1355 admissions studying the effectiveness of addiction consult services, acute care utilization (hospitalizations and emergency department [ED] visits) and mortality (all-cause and overdose deaths) among patients hospitalized with opioid use disorder found that in the year after discharge, patients receiving the intervention had significantly fewer ED admissions than those receiving usual care. Rates of hospitalization and death were not significantly different between groups.

**Meaning:**

In this study, an interprofessional hospital addiction consultation service reduced ED visits but not hospital admissions or mortality among patients with opioid use disorder, highlighting the need for comprehensive care that goes beyond the hospital and addresses the complex health and social needs of people who use opioids.

## Teaser

This secondary analysis of a cluster randomized clinical trial assesses whether interprofessional hospital addiction consultation services affect postdischarge acute care utilization and mortality rates among hospitalized patients with opioid use disorder (OUD).

## Introduction

The overdose public health crisis in the US continues, with more than 80 000 overdose deaths in 2024 and opioids involved in more than two-thirds of these fatalities.^1^ Individuals with opioid use have higher mortality rates than the general population, driven by causes including overdose, trauma, infections, and noncommunicable diseases.^2^ Notably, both inhospital and posthospital discharge mortality among patients with opioid use disorder (OUD) are increasing.^3,4,5^ This population also has high hospitalization rates and frequent preventable readmissions and emergency department (ED) visits.^6,7,8,9^

Despite the proven efficacy of medications for OUD (MOUD) treatment, fewer than 1 in 4 Americans with OUD report receiving MOUD in the past year.^10^ With nearly half a million patients admitted to US hospitals annually with opioid-related diagnoses,^11^ the hospital setting is critical for reaching people with OUD who are at highest risk of adverse outcomes, including those not actively seeking OUD treatment.^12^ Yet the opportunity to engage patients in OUD treatment and harm reduction services during hospitalization is often missed.^13,14,15^ Patients with OUD continue to have high rates of discharge before medically advised, acute care readmissions, postdischarge morbidity and mortality, and increased health care costs.^16,17,18,19,20,21,22^

Interprofessional addiction consult services are increasingly being adopted as a solution to integrating substance use disorder (SUD) services into the continuum of hospital care.^23^ Addiction consult services provide expert diagnosis of SUDs, counseling on treatment options, MOUD initiation and dose adjustment, psychoeducation, harm reduction, and linkage to postdischarge care.^24,25,26^ Previous studies suggest that addiction consult services are associated with lower acute care utilization and mortality after hospitalization among individuals with OUD.^27,28,29,30,31^ Members of our team previously completed a pragmatic trial evaluating the effectiveness of Consult for Addiction Treatment and Care in Hospitals (CATCH), an addiction consult service for increasing postdischarge uptake of MOUD.^32^ Patients discharged from CATCH hospitals had significantly higher odds of MOUD initiation (7.96 times as high) and 30-day MOUD engagement (6.90 times as high).^33^ In this prespecified analysis, we present secondary outcomes from the CATCH trial, focusing on acute care utilization and mortality rates 1 year after discharge.

## Methods

### Study Design and Setting

A pragmatic hybrid type 1 implementation-effectiveness trial^34^ of the CATCH addiction consult service was conducted in the New York City Health + Hospitals (H+H) public hospital system. The 6 participating hospitals were selected by H+H from their 11 total hospitals to include those with high prevalence of patients with SUD and considering their geographic location. The dual primary outcome of the trial was postdischarge initiation of MOUD and engagement in outpatient MOUD treatment. The present analysis of the study focused on the following prespecified secondary outcomes: (1) acute care utilization (ED and hospital admissions), and (2) death, unintentional drug poisoning, or overdose death. Details on the study design and methodology were previously presented,^32^ and the trial is registered (NCT03611335). The full trial protocol (Supplement 1) was approved by the New York University School of Medicine institutional review board with a waiver of consent because administrative data were retrospectively analyzed for eligible patients who were not recruited or enrolled. The H+H policy requires all patients to sign a general Health Insurance Portability and Accountability Act release at their initial presentation for care, and patients did not provide separate informed consent for receiving CATCH services. We followed the Consolidated Standards of Reporting Trials Extension (CONSORT Extension) reporting guideline for cluster randomized trials for reporting findings.^35^

CATCH program implementation followed a stepped-wedge design,^36^ with hospitals initiating CATCH between October 2018 and February 2020. Hospitals were randomized to a start date and informed of their start date at the time of randomization. Hospitals implemented CATCH sequentially at the assigned dates, separated by approximately 3 months (timeline presented in eFigure 1 in Supplement 2). Outcomes were compared for the 12 months of treatment as usual (TAU) and the initial 12 months of the CATCH intervention at each hospital. The first month of CATCH implementation at each hospital was considered transitional and excluded from the analyses.

### Eligibility Criteria

Eligible cases were adult patients (≥18 years of age) admitted to a hospital medical or surgical unit, with a length of stay of at least 1 night, and at least 1 opioid-related diagnosis (OUD, opioid poisoning, or opioid adverse effects) among the admission or discharge diagnoses, based on *International Statistical Classification of Diseases, Tenth Revision (ICD-10)* diagnostic codes. Patients receiving MOUD in the 30 days before admission were excluded. We also excluded dual-eligible individuals enrolled in both Medicaid and Medicare, as their outcomes would not be completely captured in Medicaid data, which was the primary data source. The study conditions were defined based on implementation of CATCH at the hospital level, and receipt of CATCH services was not required for eligibility. Admissions in which the patient was deceased prior to discharge were not eligible, given our focus on postdischarge outcomes. For individuals with multiple admissions meeting eligibility criteria during the study period, only their last admission was included in the analysis (226 patients had multiple admissions during the study period). Patients with an admission during the TAU period who had previously been admitted to a hospital with CATCH implemented were excluded (n = 4) due to the risk of contamination through prior exposure to the intervention. Due to differences in the measurement period and eligibility criteria, the sample size for this analysis differs from that of the primary outcome analysis.

### Intervention

CATCH is an interprofessional addiction consult service, with teams consisting of 3 providers: medical clinician (physician or nurse practitioner), social worker or addiction counselor, and peer counselor. Each hospital received funding to support 3 CATCH teams, and was required to have at least 2 fully staffed teams before randomization. Teams were based in the psychiatry department at each facility, and at 3 hospitals, the lead physician was a psychiatrist.^37^ CATCH consultations could be ordered by the patient’s primary team. CATCH provided comprehensive SUD care, including diagnosis, treatment recommendations, counseling, overdose prevention education (with take-home naloxone kits), and linkage to postdischarge SUD treatment. Patients without a treatment linkage at the time of discharge could be referred to short-term bridge clinics. While the trial evaluated outcomes only among patients with an opioid-related diagnosis, CATCH also provided care to patients with alcohol and other types of nontobacco SUDs.

### Data Sources and Measures

Data included in the analysis were informed by Medicaid paid claims data (hereafter referred to as Medicaid data), electronic health record (EHR) data from the H+H clinical data warehouse, and New York City Department of Health and Mental Hygiene (NYC DOHMH) Bureau of Vital Statistics (BVS), and NYC DOHMH Bureau of Alcohol and Drug Use Prevention, Care, and Treatment (BADUPCT) unintentional overdose mortality surveillance.

Demographic and clinical characteristics, including sex, age, race and ethnicity, clinical department, admission and discharge dates, and billing diagnoses^33^ were extracted from Medicaid and EHR data to create the analytic dataset, in which individuals were assigned a unique study identifier. Demographic characteristics were self-reported at the time of Medicaid application. Race and ethnicity data are reported herein to more fully characterize the cohort. Diagnoses of chronic medical and psychiatric conditions were described for the past 3 years prior to admission, using the Chronic Condition Indicator, version 2017.1, and the Clinical Classifications Software, version 2017.1 (Healthcare Cost and Utilization Project).^38^

The following 2 outcomes 1 year after discharge were compared for eligible cases between the CATCH (intervention) period and the TAU period: acute care utilization, defined as the number of hospitalizations and ED visits; and death. Acute care utilization was derived from Medicaid data. All-cause deaths were derived from death certificate data through the BVS. Their classification as an overdose death or opioid-involved overdose death was from BADUPCT unintentional overdose mortality surveillance data,^39^ which identifies overdose deaths from cause of death codes on death certificates and links these cases to the NYC Office of the Chief Medical Examiner investigation data to identify substances involved. The *ICD-10* codes for unintentional drug poisoning (X40-X44) and mental and behavioral disorder (F11-16, and F18-19, excluding those with a fourth character position of 2 or 6) were used to classify overdose deaths based on the underlying and multiple cause of death codes. The postmortem alcohol and drug toxicology data from medical examiner data were used to determine whether the overdose death involved opioids. The detailed methods for defining overdose deaths and the list of postmortem toxicology metabolites is described elsewhere.^40,41^

A sample extracted from the EHR data was created for transfer to NYC DOHMH for matching. The sample included any adult patient admitted to the inpatient departments served by CATCH with at least 1 opioid-related diagnosis during the study period and with no activity in the EHR after 1 year postdischarge. The identifying data, including study identification number, first and last name, date of birth, date of death (if available), sex, social security number, and zip code, were sent by the research team (Y.R.-A., J.M.) to BVS, where patient records were matched to death certificate databases on exact and near matches of identifying data. Matches were reviewed using a Microsoft Access database, in which records from the 2 sources were presented side-by-side to facilitate comparison.

In a second step, BADUPCT shared with BVS the death certificate number, date of death, and information on whether a death involved opioids. Individuals with a strong match in the BVS death certificates were matched against BADUPCT data to identify overdose deaths and to determine whether they involved opioids. Subsequently, for each identified case, data were provided to the research team by BVS with the study identifier, date of death, overdose death, and opioid-involved overdose death. Using the study identifier, these variables were joined to the analytic dataset.

### Statistical Analysis

Eligible admissions during the first year following implementation of CATCH at each hospital, excluding the initial transitional month, were designated as intervention admissions. Admissions that occurred in the 1 year before CATCH implementation at each hospital were considered TAU admissions (eFigure 1 in Supplement 2).

For the acute care utilization analysis, Poisson mixed-effects regression models were used to compare the effect of the intervention vs TAU, and the models were adjusted for age, sex, time, and a random effect at the hospital level. To evaluate the effect of time (to adjust for possible underlying patterns in the outcome), we fitted models with and without the linear period variables. The models with the period variable were significantly different from the reduced models, with lower Akaike information criterion and similar Bayesian information criterion. Therefore, the results presented here are for the full model, including the period variable.

For the death analysis, time to death was defined as the number of days following the hospital discharge date. Cox proportional hazards models were used to compare mortality among the study groups, adjusted for age, sex, time since the beginning of the study, and hospital cluster effect. Survival plots were based on Cox proportional hazards models stratified by the study condition. We utilized a 2-sided significance level of α = 0.05 for all analyses. Analyses were performed for eligible patients between July 2023 and September 2024, using R statistical software, version 4.3.0 (R Project for Statistical Computing).

## Results

Among the 1355 eligible cases (Figure), most were male (968 [71.4%]; 387 [28.6%] female), and the mean (SD) age was 46.6 (12.4) years (Table 1). Overall, 451 individuals (33.3%) were identified in Medicaid data as African American or Black, 73 (5.4%) as American Indian, Asian, and Native American, 59 (4.4%) as racially diverse, and 462 (34.1%) as White; race was unknown for 310 individuals (22.9%). Almost one-third of individuals (423 [31.2%]) in the sample were identified in Medicaid data as Hispanic. While all cases had at least 1 opioid-related diagnosis, 335 (24.7%) also had an alcohol-related diagnosis, and 665 (49.1%) had a nonopioid drug diagnosis. In the 3 years prior to the hospitalization, over two-thirds of cases (940 [69.4%]) had 3 or more chronic medical conditions, while 875 (64.6%) had at least 1 serious mental illness. Median (range) length of stay in the hospital was 4.0 (1.0-241.0) days in the CATCH period and 5.0 (1.0-187.0) days in the TAU period.

**Figure.**
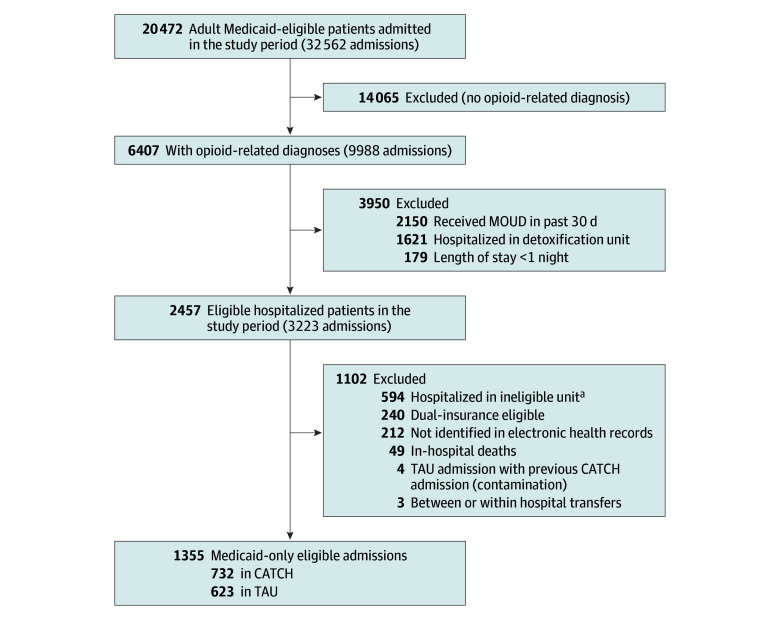
Consolidated Standards of Reporting Trials Diagram CATCH represents Consult for Addiction Treatment and Care in Hospitals; MOUD, medication for opioid use disorder; and TAU, treatment as usual. ^a^Hospitalizations in dedicated psychiatric or detoxification units, or jail or prison units, were not eligible.

**Table 1.** Demographic and Clinical Characteristics of the Study Population

Characteristic	Participants, No. (%)		
	Total (N = 1355)	TAU (n = 623)	CATCH (n = 732)
Sex			
Female	387 (28.6)	194 (31.1)	193 (26.4)
Male	968 (71.4)	429 (68.9)	539 (73.6)
Age at admission, y			
Mean (SD)	46.6 (12.4)	46.8 (12.5)	46.4 (12.4)
Median (range)	48.0 (18.0-96.0)	49.0 (18.0-96.0)	48.0 (18.0-85.0)
Race^a^			
African American or Black	451 (33.3)	205 (32.9)	246 (33.6)
American Indian, Asian, or Native American^b^	73 (5.4)	34 (5.5)	39 (5.3)
Racially diverse	59 (4.4)	33 (5.3)	26 (3.6)
White	462 (34.1)	226 (36.3)	236 (32.2)
Unknown	310 (22.9)	125 (20.1)	185 (25.3)
Ethnicity^a^			
Hispanic	423 (31.2)	200 (32.1)	223 (30.5)
Not Hispanic	685 (50.6)	322 (51.7)	363 (49.6)
Unknown	247 (18.3)	101 (16.2)	146 (19.9)
Length of stay, d			
Mean (SD)	10.8 (19.4)	11.2 (20.1)	10.4 (18.8)
Median (range)	5.0 (1.0-241.0)	5.0 (1.0-187.0)	4.0 (1.0-241.0)
Any alcohol diagnosis	335 (24.7)	148 (23.8)	187 (25.5)
Any nonopioid drug diagnosis	665 (49.1)	293 (47.0)	372 (50.8)
Any chronic medical condition^c^			
None	88 (6.5)	39 (6.3)	49 (6.7)
1 or 2	327 (24.1)	139 (22.3)	188 (25.7)
≥3	940 (69.4)	445 (71.4)	495 (67.6)
Any chronic mental illness^d^			
No	310 (22.9)	155 (24.9)	155 (21.2)
Yes	1045 (77.1)	468 (75.1)	577 (78.8)
Any serious mental illness^d^			
No	480 (35.4)	231 (37.1)	249 (34.0)
Yes	875 (64.6)	392 (62.9)	483 (66.0)
Hospitalizations^e^			
Mean (SD)	2.9 (4.8)	2.7 (4.4)	3.0 (5.0)
Median (range)	1.0 (0.0-46.0)	1.0 (0.0-32.0)	1.0 (0.0-46.0)
No visits	502 (37.0)	220 (35.3)	282 (38.5)
1 or 2 Visits	359 (26.5)	176 (28.3)	183 (25.0)
≥3 Visits	494 (36.5)	227 (36.4)	267 (36.5)
Emergency department visits^e^			
Mean (SD)	5.3 (15.5)	4.9 (14.0)	5.7 (16.6)
Median (range)	1.0 (0.0-284.0)	1.0 (0.0-221.0)	1.0 (0.0-284.0)
None	520 (38.4)	251 (40.3)	269 (36.7)
1 or 2	396 (29.2)	176 (28.3)	220 (30.1)
≥3	439 (32.4)	196 (31.5)	243 (33.2)
Death^e^			
All-cause	113 (8.3)	52 (8.3)	61 (8.3)
Overdose	34 (2.5)	16 (2.6)	18 (2.5)
Opioid overdose	28 (2.1)	13 (2.1)	15 (2.0)
Time to death following hospital discharge, d			
Mean (SD)	129.3 (101.3)	121.8 (99.0)	135.6 (103.6)
Median (range)	118.0 (1.0-360.0)	117.0 (1.0-360.0)	121.0 (1.0-360.0)

Abbreviations: CATCH, Consult for Addiction Treatment and Care in Hospitals; TAU, treatment as usual.

^a^ Race and ethnicity classifications are based on patient self-report from Medicaid data.

^b^ These classifications have been grouped together to avoid reporting populations less than 10.

^c^ Chronic conditions were measured using the Chronic Condition Indicator, version 2017.1 and the Clinical Classifications Software, version 2017.1.

^d^ Chronic mental illness includes mood disorders, bipolar disorders, schizophrenia, and other mental illness conditions. Serious mental illness is limited to manic episodes, bipolar disorder, major depressive disorder, schizophrenia, and other psychotic disorders.

^e^ Twelve months after discharge.

During the 12-month hospital postdischarge follow-up, 835 patients (61.5%) had at least 1 ED visit or hospitalization. The median (range) number of hospitalizations was 1.0 (0.0-46.0), and the median (range) number of ED visits was also 1.0 (0.0-284.0). After adjusting for age, sex, time, and the cluster effect at the hospital level, the incidence rate ratio for ED visits was 0.79 (95% CI, 0.72-0.88; *P* < .001) for CATCH compared with TAU. The incidence rate ratio for hospitalizations was 0.99 (95% CI, 0.87-1.13; *P* = .90) for CATCH compared with TAU (Table 2).

**Table 2.** Effect of CATCH Intervention (vs TAU) on Acute Care Utilization and Death in the Study Population

Outcome	IRR or HR (95% CI)	*P* value
Acute care utilization		
Hospitalization	IRR: 0.99 (0.87-1.13)	.90
Emergency department visit	IRR: 0.79 (0.72-0.88)	<.001
Death		
All-cause	HR: 1.14 (0.98-1.92)	.65
Overdose	HR: 1.06 (0.41-2.74)	.79
Opioid-involved overdose	HR: 1.03 (0.36-2.91)	.92

Abbreviations: CATCH, Consult for Addiction Treatment and Care in Hospitals; HR, hazard ratio; IRR, incidence rate ratio; TAU, treatment as usual.

During the same period, there were 113 all-cause deaths, representing 8.3% of the study sample. Among these deaths, 34 (30.1%) were overdose deaths, of which 28 (82.4%) involved opioids (Table 1). Among the all-cause deaths, 48 (42.5%) occurred within 90 days after hospital discharge. The median (range) age of decedents was 55.0 (21.0-82.0) years (mean, 51.1 years), and 29 (25.7%) were women. Among decedents, 39 (34.5%) of the individuals were identified in Medicaid data as African American or Black, 11 (9.7%) as American Indian, Asian, Native American, or racially diverse (grouped together to avoid reporting populations less than 10), 39 (34.5%) as White, and 24 (21.2%) had an unknown race; 38 (33.6%) were identified in Medicaid data as Hispanic. After adjusting for age, sex, time, and hospital cluster effect, the hazard ratio for all-cause mortality in CATCH compared with TAU was 1.14 (95% CI, 0.98-1.92; *P* = .65). Similarly, the hazard ratios for overdose mortality (1.06; 95% CI, 0.41-2.74; *P* = .79) and opioid-involved overdose mortality (1.03; 95% CI, 0.36-2.91; *P* = .92) did not reach statistical significance (Table 2). eFigure 2 in Supplement 2 presents survival probability plots for all-cause, overdose, and opioid-involved overdose deaths.

## Discussion

This prespecified secondary analysis of a cluster randomized clinical trial found high overall rates of acute care utilization in the year after hospital discharge among hospitalized patients with opioid use who were not receiving MOUD prior to admission in 6 NYC public hospitals. The CATCH interprofessional addiction consult service reduced 1-year postdischarge ED visits. However, no significant differences were detected between CATCH and TAU in 1-year postdischarge hospital admissions or in all-cause deaths or overdose deaths.

Our findings align with previous studies showing lower rates of ED visits among patients who received hospital addiction consult or services.^23,28,29^ Nonetheless, even during the CATCH period, the percentage of patients with at least 1 ED visit or hospitalization in the year after discharge was notably high at 61.5%, reflecting the overall elevated rates of acute care utilization among patients with OUD.^30,42,43^ Although a positive association between addiction consultation and lower postdischarge hospitalizations has been described,^29,31^ no significant effect on postdischarge hospital admissions has been found.^23,42,44,45,46^ Further investigation of the leading causes of postdischarge ED visits and readmissions could help characterize the health needs and drivers of acute care utilization in this population.

In the present study, mortality was high (8.3%) in the year following hospitalization, and 42.5% of deaths occurred within the first 90 days after discharge, similarly reported in previous research.^4,5,18,47^ Consistent with prior studies,^18^ while overdose deaths were prevalent, less than one-third of all deaths (30.1%) were attributable to overdose. Our findings reflect the multiple medical and psychiatric comorbidities of this complex patient population, as well as the emergence of fentanyl as a powerful driver of overdose deaths in New York City during the study period.^48^

Prior studies have demonstrated that retention in treatment is positively associated with acute care utilization and mortality rates,^49,50^ and a prior analysis by members of our team did not show significant differences between CATCH and TAU in 6-month MOUD retention.^33^ Inpatient addiction consult services are fundamentally time-limited hospital-based interventions, with limited ability to influence longer-term treatment and health care utilization outcomes. In the hospital, these services have clear benefits, including improved withdrawal management, delivery of evidence-based and patient-centered care for substance use,^51,52^ and integration of harm reduction interventions,^53^ and they improve rates of initiation and 30-day engagement in postdischarge MOUD treatment.^33^ Yet most hospitalizations are brief, and the median length of stay was even lower in CATCH (4 days) than in TAU (5 days). After patients leave the hospital, they face numerous barriers to continuing MOUD, medical, and psychiatric treatment,^54,55^ and difficulty navigating systems of care,^56^ which may underlie the overall low rates of retention in MOUD treatment.^57^ More robust support during the transition from hospital to community, such as postdischarge patient navigation,^30^ could improve early treatment engagement.^58,59^ Yet to meaningfully improve health outcomes, broad program and policy solutions that remove structural barriers impeding access to health and mental health services^60^ and improve options for patient-centered MOUD treatment^54,55,61,62,63,64^ are needed. Improving access to and quality of existing treatment programs (eg, patient-centered methadone take-home schedules, evidence-based MOUD doses, integrating harm reduction) are important steps to improving retention.^54,55,58,60,65,66^ Offering a greater array of options for MOUD treatment, including bridge clinics and other low-barrier treatment approaches, could help address these challenges and warrant further study.^67,68,69^

### Limitations

While our study has multiple strengths, including being a rigorous pragmatic trial conducted in safety-net hospitals, there are limitations to the interpretation of our findings. First, our analysis was confined to individuals identified in Medicaid data. Approximately 70% of hospitalized patients in the H+H system have Medicaid^70^; thus, our study captures the majority of eligible cases. However, these findings may not be generalizable to individuals with different insurance, including those who are Medicaid and Medicare dual-eligible, privately insured, or uninsured. Moreover, the Medicaid data may not capture all health care utilization if the individual had a lapse or loss of coverage or used out-of-state services during the study period. Second, we limited the sample to the last eligible admission, as one of the study outcomes was terminal, and this approach also mitigated possible contamination by excluding a small number of patients who were hospitalized in a CATCH-active hospital and then later hospitalized in a TAU hospital. There were individuals in the sample with more than 1 CATCH admission, and a possible dose-related impact of the intervention is unmeasured here. Third, administrative data sources are frequently missing data about patient characteristics, including race and ethnicity, or have unreliable capture of important social determinants of health, including homelessness, that may be associated with our outcomes of interest. Fourth, although our analyses included a temporal variable to account for possible underlying secular patterns, some changes in the hospital or postdischarge environment may remain unmeasured. Fifth, the onset of the COVID-19 pandemic during the study period affected hospital operations and access to care. Although a temporal variable was included in the analysis, outcomes may still have been influenced by the pandemic. Sixth, even though the sample was relatively large, the study was not powered to detect statistically significant differences in the rare outcome of mortality. Seventh, due to the study design, the intervention allocation was conducted at the hospital level, resulting in an intervention sample that likely included patients who did not receive CATCH consults. In addition, although our study population was substantially diverse in terms of race and ethnicity and reflective of the target population in New York City, outcomes may vary in other geographic areas and patient populations.

## Conclusions

This secondary analysis of a pragmatic cluster randomized trial demonstrated that the CATCH interprofessional addiction consult service was effective in reducing ED admissions in the year following hospital discharge among patients with OUD. Nonetheless, our study population continued to have elevated rates of acute care utilization and high rates of all-cause mortality and overdose deaths that were not significantly different between usual care and the CATCH intervention. These findings illustrate the urgency of developing and deploying comprehensive strategies that extend beyond hospital-based interventions, such as outpatient and community-based services as well as policy and services reforms, to improve health care and treatment for people with OUD.

## Notes

Supplement 1.
Trial Protocol

Supplement 2. **eFigure 1**. Timeline of study periods and outcome measures **eFigure 2.** Survival probability plots for all-cause, overdose, and opioid-involved overdose deaths

Supplement 3.
Data Sharing Statement
