# Supplementary material for: Addiction Consult Services, Mortality, and Acute Care Utilization in Inpatients With Opioid Use Disorder: A Secondary Analysis of a Cluster Randomized Clinical Trial
Source: JAMA Netw Open. 2025 Aug 6;8(8):e2525222. doi: 10.1001/jamanetworkopen.2025.25222 (PMC12329607; doi:10.1001/jamanetworkopen.2025.25222)
Supplement: Supplement 1. — Trial Protocol [file jamanetwopen-e2525222-s001.pdf]

Protocol for Effectiveness of the Consult for Addiction Treatment and Care in Hospitals (CATCH) model for engaging patients in opioid use disorder treatment: Pragmatic trial in a large municipal hospital system

s18-00096

Principal Investigator: Jennifer McNeely, MD, MS
Department of Population Health
NYU Grossman School of Medicine

Funding: NIH/NIDA

TABLE OF CONTENTS

1. Purpose of the Study and Background
    1.1. Background
    1.2. Brief overview
    1.3. Specific aims
    1.4. Study design
    1.5. Study sites
    1.6. Evaluation framework: RE-AIM
    1.7. Primary and secondary outcome measures
    1.8. Power calculation and sample size

2. Characteristics of the research population
    2.1. Overview of the research population
    2.2. Demographic characteristics
    2.3. Inclusion and exclusion criteria
    2.4. Vulnerable subjects

3. Methods and procedures
    3.1. Sources of materials
    3.2. Procedures for data collection
    3.3. Data analysis
    3.4. Data monitoring
    3.5. Data storage and confidentiality

4. Risk/benefit assessment
    4.1. Importance of the knowledge to be gained
    4.2. Risk
    4.3. Protections against risks
    4.4. Potential benefits to the subjects

5. Subject identification, recruitment and consent
    5.1. Method of subject identification and recruitment
    5.2. Process of consent
    5.3. Subject capacity
    5.4. Consent forms and Documentation of Consent
    5.5. Costs to the subject
    5.6. Payment for participation

6. Research Team

Effectiveness of the Consult for Addiction Treatment and Care in Hospitals (CATCH) model for engaging patients in opioid use disorder treatment: Pragmatic trial in a large municipal hospital system
9.7.2023

1. Purpose of the Study and Background

1.1. Background

The US is in the midst of an opioid crisis that is having an unprecedented impact on population health.[1, 2] Individuals with opioid use disorder (OUD) have disproportionately high rates of hospitalization,[3] and low rates of addiction treatment.[4] Hospitalization presents an opportunity to engage this population in effective treatment, thereby reducing their risk of acute care utilization, overdose (OD) death, and poor health and social outcomes, and yet in most hospitals OUD is not addressed. Addiction consult models offer a potential solution, by using multidisciplinary teams to evaluate patients, start addiction treatment in the hospital, and connect patients to post-discharge care.

A *Consult for Addiction Treatment and Care in Hospitals (CATCH)* intervention is being introduced into the New York City Health and Hospitals (H+H) system. H+H is the largest municipal hospital system in the US, and its 70 locations throughout the five boroughs of NYC provide care to over one million New Yorkers each year. In 2018, CATCH will be implemented in six H+H hospitals, with the intention to spread these services throughout the H+H system. CATCH Teams will evaluate hospital inpatients to diagnose OUD, start medication for addiction treatment (MAT) when indicated, and directly link patients to post-discharge treatment.

1.2. Brief overview

This pragmatic clinical trial seeks to evaluate the effectiveness of the CATCH intervention as a strategy for engaging patients with OUD in addiction treatment. The CATCH intervention uses a multidisciplinary addiction consult team, working in the hospital inpatient setting, to evaluate patients with substance use disorders (SUD), start MAT while patients are hospitalized, and connect patients to post-discharge treatment. Beginning in 2018, CATCH will be implemented in six NYC H+H hospitals; this study takes advantage of the rollout of the intervention to study the effectiveness and implementation of CATCH as delivered by clinical staff and at scale.

The study sites are the 6 hospitals that have been selected by H+H for implementing CATCH services. A stepped-wedge cluster randomized trial design will determine the impact of CATCH on opioid treatment outcomes in comparison to usual care for an approximately 12-28 month control period, followed by an approximately 12-41 month intervention period, at each site. Consistent with a pragmatic trial approach, the research is conducted in collaboration with the health system, in real-world settings and at scale. The design applies random sequential assignment of sites to CATCH until all sites have converted, and utilizes existing administrative data to evaluate outcomes. This hybrid effectiveness-implementation study (Type 1)[5] focuses primarily on the effectiveness of this new intervention for increasing initiation and engagement in treatment, while also measuring implementation outcomes to inform its later dissemination to other health systems. Using the RE-AIM framework, we will evaluate CATCH on dimensions that define its potential for public health impact.

1.3. Specific aims

The study has one primary and three secondary aims:

Aim 1 (Primary aim): Evaluate the effectiveness of CATCH in increasing MAT initiation and engagement among patients with OUD. CATCH will start MAT while patients are hospitalized, but because meaningful outcomes require linking patients to ongoing care, our measures are based on receipt of post-discharge MAT.

Hypothesis 1a (H1a): Patients hospitalized during the CATCH period will have higher rates of treatment initiation, defined as initiating MAT outpatient treatment within 14 days of discharge.

Hypothesis 1b (H1b): Patients hospitalized during the CATCH period will have higher rates of treatment engagement, defined as having 2 or more additional MAT services within 30 days of treatment initiation.

Aim 2: Assess the effectiveness of CATCH for increasing MAT retention in patients with OUD. Hypothesis 2 (H2): Patients hospitalized during the CATCH period are more likely to be continuously retained in treatment for at least 6 months, in comparison to patients hospitalized during the usual care period,.

Aim 3: Compare the frequency of acute and ambulatory care utilization and overdose deaths, and their associated costs, among patients with OUD hospitalized during the CATCH period versus usual care, and assess the economic value of CATCH with respect to MAT initiation and retention. Hypotheses: Patients in the CATCH period will have (H3a) lower rates of ED and hospital admission, and (H3b) lower rates of overdose death, in the 12 months following the index hospitalization

Aim 4: Evaluate implementation outcomes at CATCH sites using a mixed methods approach to assess additional RE-AIM elements: *Reach* – proportion of eligible patients reached by CATCH; *Adoption* – utilization of CATCH by medical staff; *Implementation fidelity* – characteristics of the intervention as delivered to the target population. A mixed-methods approach, guided by the Consolidated Framework for Implementation Research (CFIR) and gathering data from patients and staff, will provide insights into barriers and facilitators of full-scale implementation.

1.4. Study design

A stepped-wedge cluster randomized trial design will determine the impact of CATCH on OUD treatment outcomes in comparison to usual care, at each site. The design applies random sequential assignment of sites to CATCH until all six sites have converted, and primarily utilizes administrative data, (including Medicaid claims and electronic health record data), to evaluate outcomes. Interviews with CATCH staff and patients are included to inform implementation outcomes.

This is a Hybrid Type 1 study[5] in which the primary focus is on the effectiveness of the CATCH intervention, but implementation process measures are also tracked. Distinct from pure effectiveness trials, hybrid studies aim to also answer the question "What are the barriers and facilitators to 'real-world' implementation of the intervention?"[5, 6] Hybrid Type 1 designs are appropriate when there is strong face validity and at least indirect evidence supporting the intervention, and it is associated with minimal risk. While a small RCT and multiple observational studies indicate that addiction medicine consult teams may be effective for increasing OUD treatment, many questions remain before this approach can be recommended for broad dissemination. This research will provide the required knowledge about effectiveness, while simultaneously collecting important information on the cost and feasibility of implementation.

Following our stepped-wedge randomized trial design, all sites are evaluated pre- and post-implementation, with start dates randomly assigned to improve causal inference.[7-9] Each of the 6 participating hospitals will be randomly assigned to one of 6 start times; start times are separated by approximately 3 months. Each site has an approximately 12-28 month TAU observation period, followed by an approximately 12 to 41-month CATCH intervention period; the duration of TAU and CATCH periods are dependent on the site's start time. Our main study outcomes are measured at 6 and 12 months following introduction of CATCH at each site, but administrative data will continue to be collected at all sites until the end of the study period (in the 14th Quarter of the study), thus allowing us to monitor the maintenance of outcomes. The primary outcome is the rate of MAT initiation and engagement following hospital discharge, measured in Medicaid claims data.

Figure 1. Stepped-wedge design with 6 sites

Group	Study Period													
	Q1	Q2	Q3	Q4	Q5	Q6	Q7	Q8	Q9	Q10	Q11	Q12	Q13	Q14
**Site 1**														
**Site 2**														
**Site 3**														
**Site 4**														
**Site 5**														
**Site 6**														

Legend: Each box represents 3 mos [white box] =Treatment as usual measures [grey box] =CATCH intervention measures

1.5. Study sites

The six study sites are NYC Health+Hospitals (H+H) hospitals in New York City.

1. *Bellevue Hospital* (462 First Ave., New York, NY 10016).
2. *Coney Island Hospital* (2601 Ocean Parkway, Brooklyn, NY 11235)
3. *Elmhurst Hospital Center* (79-01 Broadway, Elmhurst, NY 11373)
4. *Lincoln Medical and Mental Health Center* (234 E. 149th St., Bronx, NY 10451)
5. *Metropolitan Hospital* (1901 1st Ave., New York, NY 10029)

6. *Woodhull Medical and Mental Health Center* (760 Broadway, Brooklyn, NY 11203)

1.6. Evaluation framework

Our study compares the impact of the CATCH intervention versus TAU on OUD treatment initiation, engagement, and retention, while assessing its adoption and implementation in practice. The assessment plan is organized by the RE-AIM

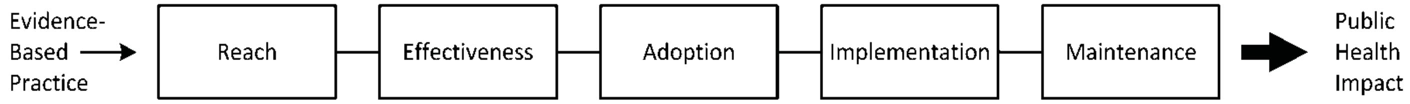

framework, which is one of the most widely applied frameworks for evaluating health behavior change programs.[10] RE-AIM is appropriate for a pragmatic trial because it offers a framework for measuring the impact of an intervention *as delivered in real world environments*. Assessment of the five domains of RE-AIM provides an evaluation of a program's potential public health impact. In our study, **Effectiveness** is the rate of OUD treatment initiation and engagement (primary outcome). **Reach** is the proportion of patients who receive CATCH services. The product of Reach*Effectiveness signifies the individual-level impact of the intervention. Implementation outcomes, measured in the intervention condition only, include: **Adoption-**the utilization of the CATCH teams by clinical staff, as measured by the rate of referrals of patients with OUD; and **Implementation fidelity-** the ability of the CATCH teams to identify and reach their target population, and deliver high-quality MAT. We have limited ability to study Maintenance in the time-limited context of this study, but the stepped-wedge design will allow us to measure Reach and Effectiveness for up to approximately 41 months following implementation of the CATCH intervention.

While the overarching framework guiding our evaluation is based on RE-AIM, we will use mixed methods to explore in-depth the organizational (organizational readiness, culture, priorities), individual (attitudes, norms), and intervention characteristics (complexity, relative advantage) of CATCH, as defined by the Consolidated Framework for Implementation Science (CFIR). These measures provide additional insights into the fidelity of the intervention as delivered, potential barriers and facilitators of its full-scale implementation, and behavioral and structural factors that may explain its effectiveness.

1.7. Primary and secondary outcome measures

Outcome measures are specified in Table 1. The primary outcomes, addressed in Aim 1, are MAT initiation and engagement. Treatment initiation is defined as having an outpatient MAT encounter (in a MAT program, office-based MAT, or bridge clinic) within 14 days following hospital discharge. Engagement is defined as having two encounters in an outpatient MAT program or, for office-based treatment, filling two prescriptions for buprenorphine or naltrexone, or receiving one prescription that covers at least 28 of the first 30 days following treatment initiation. MAT programs are outpatient addiction treatment programs that provide methadone and/or buprenorphine maintenance treatment.

Outcome measures are mainly assessed using administrative data from Medicaid claims, the H+H clinical data warehouse, and DOHMH overdose data. These data sources are further detailed in Section 3.1. *Medicaid claims* as the primary data source. Medicaid claims have data from each visit to a MAT program, and for filled prescriptions (including # of days of treatment prescribed). The supplemental data source is the H+H clinical data warehouse, which can be used to identify patients who are *not* covered by Medicaid (incl. Medicare and uninsured) who had an OUD diagnosis or received MAT during the hospital stay. The *H+H clinical data warehouse* is a secondary data sources, and captures all patients who receive addiction treatment within the H+H treatment system.

Table 1. Outcome measures by specific aim

Specific Aim	Definition	Primary Data Source (Secondary Data Source)
**Aim 1: Treatment initiation and engagement**		
Treatment initiation	Outpatient MAT encounter w/in 14 days of hospital discharge (def. MAT program or office-based MAT)	Medicaid claims (H+H clinical data warehouse)
Treatment engagement	Receipt of 2+ additional MAT services w/in 30	Medicaid claims

	days of initiation<br>(def. MAT program encounters or prescriptions for MAT)	(H+H clinical data warehouse)
**Aim 2: Treatment retention**		
Rate of treatment retention	Continuous retention in treatment for 6 months (def. MAT program visits and/or MAT Rx for ≥ 80% of days)	Medicaid claims<br>(H+H clinical data warehouse)
**Aim 3: Acute care utilization and OD deaths**		
Acute care	Hospital and ED admissions in 6 months and 12 months following discharge	Medicaid claims<br>(H+H clinical data warehouse)
OD death	Poisoning death involving opioid(s)	DOHMH overdose data<br>(Medicaid claims)
**Aim 4: Implementation outcomes**		
Reach	Received any CATCH service(s)	H+H clinical data warehouse
Adoption	Referrals made to the CATCH by clinical staff	H+H clinical data warehouse
Implementation fidelity	Ability to reach target population and deliver MAT; barriers and facilitators	Interviews w/ CATCH staff and patients with OUD; CATCH program data

Qualitative interviews to assess implementation outcomes (Aim 4), will explore organizational (organizational readiness, culture, priorities), individual (attitudes, norms), and intervention characteristics (complexity, relative advantage), as defined by the Consolidated Framework for Implementation Science (CFIR). CFIR will guide qualitative interviews with staff and patients at baseline and post intervention. Interview guides will include COVID-19-related questions to gather information for our qualitative analysis on how the pandemic affected treatment access and care of patients as well as the services offered by CATCH hospital staff members.

The economic analysis will incorporate both acute and ambulatory care costs to assess the economic impact of the CATCH intervention. The CATCH intervention could reveal significant implications for broader health economic outcomes, such as patients' health-related quality of life and changes in non-healthcare resource usage (e.g., unemployment expenses) post-discharge. Although quality of life and non-healthcare resources are not collected directly from CATCH participants, we will estimate these using published literature (e,g., health-related quality of life) and public data sources (NY state unemployment statistics) within an economic evaluation simulation model. Acute care utilization measures are specified in Table 1. Additional measures of ambulatory care and SUD treatment utilization are captured in Medicaid claims data, and measured for 6 and 12 months following hospital discharge, and include:

- Outpatient medical visits, including primary care, psychiatry, and other ambulatory medical care
- SUD treatment program visits, including to outpatient and residential treatment programs

The CATCH service sees patients with other substance use disorders (SUD) in addition to OUD. Therefore, as part of our evaluation, we will be collecting descriptive data on patients with alcohol use disorder (AUD) and other SUDs; we will also explore similar aims to our OUD aims (Aims 1-4) with these populations. Exploratory measures for AUD treatment and outcomes are collected from chart review and include:

- Orders for medications to treat AUD during hospitalization
- Prescriptions for medications to treat AUD medications
- Emergency department and primary care visits

Procedures for the AUD chart reviews are described in Section 3.1.

1.8. Power calculation and sample size

Study outcomes are assessed using administrative data and applying the case definition specified in Section 2.3. We anticipate that approximately 9,000 cases (4,500 in the treatment as usual period, 4,500 in the intervention period) will qualify for inclusion in the analysis. Simulations were used to assess the statistical power to detect a range of effect sizes

for the probability of initiation over a 12-month period. Based on pilot data from Medicaid claims, we expect that 7% of the individuals under treatment as usual (TAU) will initiate MAT (range is 4-9%). We wish to be able to detect an increase in the initiation rate of 15 to 25 percentage points, and pilot data indicates that this is attainable. We conservatively assume that the variance of the cluster-level random effect on the logistic scale is approximately 0.16, which is equivalent to an ICC of 0.05. In the absence of pilot data to estimate the ICC, 0.05 is a large and therefore conservative assumption; the true ICC is likely smaller, and thus our proposed design should yield even higher statistical power.

In simulations, 1000 iterations were used to estimate the power. For each iteration, a generalized linear mixed model was fit to randomly generated data and p-values were assessed to determine if the effect was detected. With six sites of at least 700 patients per site, we have power to detect these effects using a two-sided, 0.05-level test. Specifically, we have 80%, 95%, and 99% power to detect effect sizes of 0.13, 0.18, and 0.23, respectively; these effect sizes translate to a treatment initiation rate in the intervention group of 20%, 25%, and 30%, respectively. Table 2 shows power for a range of initiation rates in the TAU period and the CATCH period; the proposed sample size yields adequate power for nearly every combination.

**Table 2** POWER N > 700		CATCH rate					
		0.20	0.25	0.3	0.35	0.4	0.45
TAU rate	0.05	95%	99%	99%	99%	99%	99%
	0.1	57%	80%	96%	98%	99%	99%
	0.15	21%	48%	67%	89%	97%	99%

Up to 120 individuals (60 staff, 60 patients) will be enrolled in the study, and will participate in qualitative interviews as described below.

2. Characteristics of the research population

2.1. Overview of the research population

CATCH services are delivered by clinical staff as part of regular care. Our study retrospectively analyzes administrative data for patients with OUD who have been hospitalized at the participating sites. These patients are not recruited or enrolled. The H+H policy is for all patients to sign a general HIPAA release when they first present for care. Patients will not be asked to provide separate informed consent for receiving CATCH services.

We will conduct qualitative interviews to assess implementation outcomes with up to 60 CATCH staff and up to 60 patients (total N=120). A purposeful sampling approach will be used to select participants with a variety of demographic characteristics, and staff selected for interviews will include individuals providing each of the CATCH team roles (i.e., medical providers, addiction counselor/social workers, and peers). Patients will be divided approximately equally between those who did and did not receive CATCH services. Individuals who participate in research interviews will provide informed consent for participation and will be enrolled in the study. Baseline interviews are conducted with CATCH staff (approximately 5/site). Post-implementation interviews are conducted approximately 9-12 months after introduction of CATCH for CATCH staff (approximately 5/site), and approximately 12 – 18 months after introduction of CATCH with patients (approximately 10/site), as summarized in Table 3 below. Distribution of staff interviews at baseline and post-implementation may vary depending on participants' availability and staffing levels at each site, but will be approximately 5 in each case. If an in-person research visit is not feasible (for example, due to travel restrictions related to COVID-19), staff interviews may also be conducted via Webex or phone. In such cases, only audio (and not video) will be recorded and saved.

Table 3. Number of participants participating in interviews at each study site.[*]

Site:	Bellevue (N participants)	Coney Island (N participants)	Elmhurst (N participants)	Lincoln (N participants)	Metropolitan (N participants)	Woodhull (N participants)	TOTAL

Staff baseline interviews	5	5	5	5	5	5	30
Staff post implementation interviews	5	5	5	5	5	5	30
Patient post-implementation interviews	10	10	10	10	10	10	60
**TOTAL**	20	20	20	20	20	20	120

[*] Distribution of staff interviews at baseline and post-implementation may vary depending on participants' availability and staffing levels at each site, but will be approximately 5 in each case.

2.2. Demographic characteristics

The cases identified through administrative data consists of individuals with OUD who are hospitalized at one of the six study sites. We anticipate that the characteristics of this population will vary by site, given the differences in patient populations served by each of the participating hospitals. Overall, we expect that cases will have the following demographic characteristics: 28% female; 14% Hispanic; 24% Black, 37% White, 1% Asian, 38% mixed race/other; 4% age 18-25, 33% age 26-45, 55% age 46-65, 7% >65 years.

Characteristics of individuals who are enrolled in the study and participate in interviews will reflect the groups from which they are drawn, and are not expected to vary substantially according to study site. Our purposive sampling approach will seek to enroll participants with a range of demographic characteristics. No one will be excluded from participation on the basis of gender, race, or ethnicity. The expected demographic characteristics of participants are indicated below in Table 4, though the actual demographic characteristics of enrolled individuals may differ.

Table 4. Expected demographic characteristics of the population enrolled for participation in interviews

Groups	Staff-Medical providers<br>N	Staff-Counselors<br>N	Staff-Peers<br>N	Patients<br>N
TOTAL	20	20	20	60
Sex				
Female	12	12	10	17
Ethnicity				
Hispanic	2	8	2	8
Race				
Black	4	8	7	14
White	14	8	7	22
Asian	2	0	0	1
Multi/Other	0	4	6	23
Age group				
18-25	0	0	3	3
26-45	10	10	7	20
46-65	10	10	7	33
>65	0	0	3	4

2.3. Inclusion and exclusion criteria

<u>Case definition for inclusion in the analysis of administrative data:</u>

1. Adult patients (≥18 years);
2. Hospitalized for at least 1 night on an inpatient service (not including intensive care or prison units);

3. Admission or discharge diagnosis (based on ICD-10 codes) of opioid use disorder or opioid poisoning.
Cases are excluded if they received MAT (buprenorphine, methadone maintenance treatment, or naltrexone) in the 30 days prior to admission. Cases included in the intervention condition must have been admitted on a date following implementation of CATCH.

Inclusion and exclusion criteria for interview participants:
Inclusion criteria:

- Age 18 years or older
- Hospitalized with ICD-10 admission or discharge diagnosis of opioid use disorder or opioid poisoning.

Exclusion criteria:

- Lack of fluency in English, unable to provide informed consent.

Based on these inclusion and exclusion criteria, cases admitted to hospital units that are not served by the CATCH program (i.e. inpatient psychiatry, detoxification unit) may appear in the analytic dataset. Some of our analyses will exclude cases from these units.

Inclusion criteria for exploratory analyses:

1. Adult patients ($\geq$18 years);
2. Hospitalized for at least 1 night on an inpatient service (not including intensive care);
3. Alcohol use disorder, alcohol poisoning, other substance use disorder, adverse effects of substance use, illicit drug poisoning.

Exclusion criteria for exploratory analyses:
None

2.4. Vulnerable subjects

We do not exclude pregnant women from participation in this minimal risk study. No other vulnerable populations are enrolled.

3. Methods and procedures

3.1. Sources of materials

Administrative data

The administrative data sources are listed below, and the measures to be extracted from each data source are listed in Appendix A.

- *Medicaid claims* are the primary data source. The Medicaid claims dataset contains information on all health care contacts and paid claims, including inpatient hospitalization, emergency department visits, outpatient visits to medical or addiction treatment providers, and filled prescriptions for medication. New York University has an existing data exchange application and agreement (DEAA) with the NYS Dept. of Health that covers the use of NYS Medicaid claims data, including personal health information and Medicaid Confidential Data. The scope of the proposed research falls under this existing agreement.
- *H+H clinical data warehouse* is a supplemental data source. The clinical data warehouse contains electronic health record (EHR) data from all H+H facilities. This data may include insurance and demographic information, provider notes, diagnoses, problem list, test results (laboratory, radiology, etc.), medication orders and prescriptions, filled prescriptions from H+H pharmacies, and location of the admission. Through the NYU-H+H Clinical and Translational Science Institute, Master Data Use Agreements have been created between H+H and NYU Grossman School of Medicine. The Master Data Use Agreements govern the sharing of data for research and specify the type of data that may be shared. In addition to the Master Data Use Agreements, NYU investigators will complete data request forms that specify the data elements to be transferred.
- NYC Dept. of Health and Mental Hygiene (DOHMH) *comprehensive database of NYC overdose deaths* is a

supplemental data source. The DOHMH Bureau on Alcohol and Drug Use compiles a database that links the NYC Vital Statistics Death Registry (death certificates recording cause of death), to NYC Medical Examiner Data files that document overdose deaths and all substances involved in the death. This dataset is developed and maintained by the NYC DOHMH, and the full identified data set cannot be shared with outside investigators. The extraction of measures for this study will be performed by a DOHMH data analyst who works with Vital Statistics and Medical Examiner data files.

- *CATCH program data* are collected for measurement of intervention fidelity. It will be collected by the research team on a monthly basis, or less frequently depending on the availability of the information, for a total of 12-41 months after CATCH introduction at each site. These data includes information on staffing, preparations for the delivery of services, intervention adherence, telephonic outreach and supervision. CATCH program data will also be analyzed to measure the impact of the COVID-19 pandemic on CATCH Program activities.
- A structured review of medical records for approximately 100 patients at each study site will be conducted by the research staff. This review will assess the rate of under-coding of OUD in billing data and medical records. The review will include patients who received MAT in the hospital to determine whether these patients should have a diagnosis of OUD. Research staff will compare the chart review findings to the ICD-10 diagnostic codes used for the hospitalization to determine the frequency with which OUD is not captured in diagnostic coding. If fewer than 90% of patients identified in the chart review have an ICD-10 discharge diagnosis of OUD or poisoning, the research team will revise their process for identifying cases eligible for CATCH services.
- As part of the data quality checking process for our analytic dataset, the research team may conduct additional chart reviews to confirm details about variables outlined in the protocol. For example, we will seek to determine or confirm the eligibility of the admission location (i.e., to exclude admissions to inpatient psychiatry, detoxification units, prison units). We plan to use the H+H Epic EHR to look up hospital admissions that meet our case definition. Trained research assistant(s) will search for cases in the EHR using one or more individual identifiers from the H+H clinical data warehouse and/or Medicaid data sets. Once the case is identified in the EHR, information will be entered directly into the secure data environment hosted in the MCIT Data Center or onto the encrypted external hard drive that is used to store Medicaid data (See Section 3.5). Note that this process does not aim to identify additional patients who meet our case definition; rather, we will be collecting additional data from the charts of the patients we already identified.
- Data for the AUD exploratory measures will be directly accessed through the Epic EHR for Bellevue Hospital. The patients will be selected from a database of all patients who have a completed CATCH consult at Bellevue between 07/01/2019 – 02/29/2020 inclusive who meet our inclusion/exclusion criteria for this exploratory analysis. We will then collect data items noted in the "CATCH Exploratory Alcohol Use Disorder Chart Review Form". Information will be directly entered into a secure NYU Langone Health (NYULH) REDCap (Research Electronic Data Capture) database. After data collection is complete, all individual identifiers will be removed from the analytic dataset, and individuals will be identified by study-assigned unique identifiers.
- Only members of the research team will have access to the study data. All research data for the AUD exploratory analyses will be extracted from the parent administrative dataset and securely transferred to a REDCap database that is provided and hosted by NYU Grossman SoM Research IT. REDCap is a HIPAA compliant secure web application for building and managing databases. REDCap provides audit trails for tracking data manipulation and user activity, and automated export procedures for data downloads to statistical programs. The NYULH REDCap database will be built and maintained by study staff, with support from NYU Grossman SoM Research IT. The database will be a HIPAA compliant, locally managed storage cluster on a secure, password-protected share drive accessible only to members of the research team. Passwords will be changed on a regular basis.

Qualitative data

Interviews will be conducted with CATCH staff and patients using IRB-approved interview guides that are based on the CFIR framework. Dr. McNeely has substantial experience conducting qualitative interviews with medical staff and patients.[11, 12]

3.2. Procedures for data collection

Administrative data

Before extracting the administrative data, the investigators and data analysts will jointly develop criteria for defining each measure in the EHR. They will develop a comprehensive data dictionary, and establish a procedure for extracting these data for analysis. Measures from each data source will be extracted and cleaned by a designated data analyst who has prior experience conducting similar projects in their specified data set. Analysts will work under the supervision of the key personnel who have specific expertise utilizing the data source (Dr. Billings for Medicaid claims, Roopa Kalyanaraman Marcello for the H+H clinical data warehouse, Ellenie Tuazon, Joe Kennedy and Dr. Van Wye, for the DOHMH overdose data). In the final data set used for analysis, individuals will be identified by unique ID numbers, and any personal identifiers will be removed.

For the Medicaid claims and H+H clinical data warehouse data, the data analysts will provide a data set to the NYULH biostatisticians that includes personal identifiers that enable matching across the data sets, along with the variables specified in the study's data dictionary. To generate the overdose data set used in our planned analyses, the NYULH data analyst will identify individuals within the Medicaid and/or H+H datasets who had no activity after month 12 following hospital discharge, and provide a limited set of identifiers (which may include any of the following: name, date of birth, sex, last 4 digits SSN), derived from the H+H data set, to the DOHMH overdose data analyst. The DOHMH data analyst will use these identifiers to identify any matches in the DOHMH overdose data. After data matching is completed, and before the data are transferred to NYULH, all individual personal identifiers will be removed other than a study ID. CATCH program data will either be entered by NYULH research staff or it will be entered by CATCH staff and program administrators into an electronic form provided by the research team.

All data containing PHI will be transferred through a secure file transfer platform. Data management will be the responsibility of the biostatistics team, who will verify the integrity and completeness of the data received, and will perform additional cleaning as needed, prior to analysis. Quality of the data extracted from each administrative data set will be evaluated and monitored by the data analysts and co-investigators working with each data source. Primary responsibility for quality assurance of data extracted from each of these sources lies with Dr. Billings for the Medicaid claims data, Roopa Kalyanaraman Marcello for the H+H clinical data warehouse data, Ellenie Tuazon and Joe Kennedy, Dr. Van Wye for the DOHMH database of OD deaths.

After data are received from each data source by the biostatisticians at NYU Grossman SoM, ongoing evaluation and monitoring of its quality will be part of data management activities. They will review the data received to ensure its completeness and accuracy. They will also review the data checking or validation reports to determine the number and types of data issues and look for trends in data collection patterns within and across sites. The biostatisticians will specifically look for invalid data, missing data, data in conflict with the specifications of the study, and data in conflict with the within- and across-record logic checks. They will also review the data longitudinally for consistency and will look for outliers or data that may exceed actual and logical limitations of each data element (i.e., range checks). They will communicate all data issues and trends to the PI and other members of the study team and work with them to rectify all issues to maintain the highest possible level of data completeness and accuracy.

Qualitative data

Interviews will be audio recorded. Audio recordings of interviews will be sent to professional transcription services and transcribed verbatim. Names and other identifying information will be removed from the transcriptions.

3.3. Data analysis

3.3.a. Administrative data analysis

Analysis of the administrative data is led by the Senior Biostatistician, who will direct the analysis in collaboration with the PI, and will direct and supervise the work of one or more biostatistician(s) in carrying out the statistical programming and data management. No interim analyses are planned. The Statistical Analysis Plan attached to this protocol contains detailed information on the analysis.

Sensitivity analysis: Because substance use diagnoses are not always completely captured in diagnostic coding,[13-15] we will also perform a sensitivity analysis on a subset of approximately 100 patients from each facility who received once daily methadone or any formulation of buprenorphine-naloxone while hospitalized during the TAU period, as determined from EHR data collected in the H+H clinical data warehouse. A structured chart review will determine whether these

patients have OUD. If fewer than 90% of patients identified in the chart review have an ICD-10 discharge diagnosis of OUD or poisoning, we will revise our process for identifying cases in the administrative data.

3.3.b Qualitative data analysis
Analysis of qualitative data will be led by the PI, Jennifer McNeely, MD, MS. Coding will be undertaken using qualitative analysis software (such as Atlas.ti or NVivo) by one or more trained researchers using an a priori coding scheme based on the Consolidated Framework for Implementation Research (CFIR), and will additionally identify any emergent themes using a grounded theory approach. Findings will be discussed among the investigators to reach consensus on the main themes and any adaptations to the CATCH implementation strategy that might support its sustainability and future implementation at other sites.

3.4. Data monitoring
This study does not fit the NIH definition of a Phase 3 clinical trial or multicenter study that would require a DSMB. The study is not blinded, poses no more than minimal risk to participants, and does not enroll vulnerable populations. As such, a Data Safety Monitoring Board is not planned.

The PI (Jennifer McNeely, MD, MS) will have overall responsibility for data analysis and data management, as well as ensuring the validity, integrity and confidential nature of all data used. Dr. McNeely will be responsible for reporting to the IRB and for monitoring all aspects of the study including adherence to the study protocol, quality assurance, and adverse events. Monitoring of data quality will be conducted on an ongoing basis by the NYU research team, and will include periodic checks against the administrative data sources, (performed by the specific data analyst for each data set), to ensure reliability.

3.5. Data storage and confidentiality
Only members of the research team will have access to the study data. Passwords will be changed on a regular basis. All research data will be extracted from the parent administrative datasets. It will then be securely transferred to and stored on either 1) an encrypted external hard drive which is kept in a locked cabinet when not in use in a locked, limited access building (Medicaid data); 2) a secure data environment hosted in the MCIT Data Center that is currently a single Windows virtual machine (VM) server instance. Designated CATCH research team members access the VM Server through CyberArk under personalized service accounts. Security controls for this data environment are implemented by the MCIT Data Center (Medicaid data) 3) a HIPAA-compliant, locally-managed storage cluster on a secure, password-protected shared drive accessible only to members of the research team (data stored at NYU Langone); or 4) a HIPAA-compliant Kiteworks application where all files are encrypted in transit and at rest, and data are secured via an SSL/TLS encrypted connection (data stored at H+H Office of Behavioral Health). Digital audio recordings will be uploaded as soon as possible from when the recording was made (typically within 24 hours) to a password-protected secure storage drive. The recordings will be accessible only to members of the research team. Audio recordings will be destroyed upon completion of the analysis after transcripts have been returned to the study team and proofed. Transcripts and notes on the interviews will contain no identifying information.

4. Risk/benefit assessment

4.1. Importance of the knowledge to be gained
The CATCH intervention has the potential to turn hospitalization into an opportunity to engage patients with OUD in effective treatment, which can ultimately reduce acute care utilization and overdose deaths in a population that is at exceedingly high risk for poor health outcomes. Our study will provide the first rigorous evaluation of an addiction consult intervention as it rolls out in a large hospital system, including a comprehensive assessment of economic outcomes (i.e., cost-effectiveness). The hybrid type 1 effectiveness-implementation design provides evidence regarding the effectiveness, feasibility, and public health impact of the addiction consult model that health systems, insurers, and policymakers require.

4.2. Risk

This is a minimal risk study that primarily involves the analysis of administrative data. The administrative data sources are non-public, protected sources that are secured according to standard protections at the institutions where the data are housed and maintained: NYU (Medicaid claims), H+H (clinical data warehouse data), and DOHMH (overdose data). Risks include the disclosure or breach of individually identifiable health information by study personnel. The final data set used for our planned analyses will be de-identified. Access to research offices at NYU is restricted to research staff, and the buildings where these offices are located are secure and monitored by security staff. All electronic data are stored on a secure, password-protected shared drive that is accessible only to members of the research team.

This study poses no more than minimal risk to research participants, who are CATCH staff and patients who enroll in the study and participate in interviews. The main risk of participation is loss of confidentiality or discomfort from answering interview questions. Personal identifying information collected for the study will be kept to the minimum required to collect the specified information. We will minimize discomfort by having interviews conducted by a trained and qualified researcher, in a private space. Participants will be informed that all aspects of their participation are voluntary and that they may choose not to complete assessments or to discontinue their participation entirely, without repercussion or penalty. For staff, adequate protections will be implemented to ensure that participation in the research does not jeopardize their employment or professional standing; for patients, protections will be implemented to ensure that participation in the study does not impact their medical care. Information concerning the Certificate of Confidentiality and its implications will be provided to participants in the consent form(s).

4.3. Protections against risk

Recruitment and Informed Consent

Cases identified in administrative data: CATCH services are delivered by clinical staff as part of regular care. Our study retrospectively analyzes administrative data for patients with OUD who have been hospitalized at the participating sites. These patients are not recruited or enrolled, and a waiver of authorization is requested. The H+H policy is for all patients to sign a general HIPAA release when they first present for care. Patients will not be asked to provide separate informed consent for receiving CATCH services.

Participants in qualitative interviews: Staff and patients are recruited to participate in qualitative interviews. A purposeful sampling approach will be used to select CATCH staff with a variety of roles, demographic characteristics, and backgrounds to participate in qualitative interviews. Interviews will be conducted outside of working hours in accordance with the participant's availability and preference, and participants will receive $50 compensation for their time. Research staff will obtain verbal consent for the interviews, and written consent for audio recording. Consent documents will be in English using an IRB-approved consent form that includes name and contact information of the PI and the IRB, a description of the study, the payment schedule, a description of potential risks and benefits, a statement of confidentiality, required language regarding HIPAA, and an indication of the right to refuse or withdraw at any time without any consequence.

Data Safeguards

The data sources are all existing administrative data sets that are secured according to standard protections at the institutions where the datasets are currently housed and maintained: NYU Wagner School and NYU Grossman School of Medicine, Dept. of Population Health (Medicaid claims); H+H (clinical data warehouse data); and DOHMH (overdose data). All data containing PHI will be transferred through a secure file transfer platform. Once any required matching is completed, the research data will be de-identified by assigning anonymous study IDs to all cases. The identified research data will be destroyed, and only the de-identified data set will be used for analysis. Access to research offices at NYU is restricted to research staff, and the buildings where these offices are located are secure and monitored by security staff. All electronic data are stored on a secure, password-protected shared drive that is accessible only to members of the research team. Audio recordings are destroyed upon completion of the analysis. Data on the NYULH shared drive are stored in an off-site datacenter restricted to authorized personnel and managed by NYU Grossman SoM staff. Backups are performed nightly and replicated in disaster recovery site at an alternate location. Data is stored on secure servers existing behind the data center firewall with permission-based access. Security controls in place for all storage include,

but are not limited to, anti-virus, intrusion detection and prevention, and access logging. All systems undergo periodic patching and penetration testing.

Privacy and Confidentiality
Standard IRB-approved and HIPAA compliant measures will be used to maintain confidentiality, privacy and data security. Interviews will take place in private rooms, and participants will be informed that they are audio recorded. Audio recordings will be transferred as soon as possible (typically within 24 hours) to a secure server, and accessible only to the research staff and transcription service. The digital audio files will be destroyed upon completion of the analysis, so that the audio recordings may be referred to in the analysis if needed for clarification. Digital audio files will be deleted when data collection, transcription, proofing and analysis are completed. Transcripts will not contain names or other identifying information. All data will be analyzed and reported anonymously.

All study personnel will have taken the mandatory training required by their IRBs, including HIPAA and Patient Privacy/Confidentiality training, to ensure that they are aware of the importance of patient confidentiality and appropriate laws regarding the protection against patient privacy breaches. Procedures will be in place to ensure that all files containing patient information will be kept in locked filing cabinets and/or password-protected electronic databases. There will also be a system in place for breaches in patient privacy or other adverse events to be reported to the study PI, who will take the appropriate steps to ensure that they are documented and that there is minimal risk that it could happen again.

Adverse Events and Serious Adverse Events
No Adverse Events (AEs) or Serious Adverse Events (SAEs) are anticipated in this study. Any adverse event that may occur will be recorded with the name of the event, the relationship to study participation, the severity and resolution. Adverse event reports will be reviewed by the PI within 24 hours, and reported to the IRB within 3 days of the adverse event's occurrence, following NYU Grossman SoM reporting requirements. The PI will review all adverse events reported for the determination of seriousness, severity and relatedness. Serious adverse events (SAEs) and unanticipated problems involving risks to participants or others related to study procedures will be reported to the IRB within 24 hours, following NYU Grossman SoM reporting requirements. New information that may affect study participation will be provided to participants in a timely fashion. AEs and SAEs will be followed through resolution, stabilization or study end, and any serious and study-related AEs will be followed until resolution or stabilization, even beyond the end of the study.

A written report of any AEs will be sent to the NIDA Program Officer (PO) as part of the annual progress report. This report will include a description of the event, when it occurred, the study arm of the participant, and the outcome/resolution. If there were no AEs, a statement that no AEs occurred will be included in the progress report. SAEs will be reported to the NIDA PO by email, and to the NYU Grossman SoM IRB, within 24 hours of the event. This initial 24-hour notification will include a brief explanation of the SAE and when it occurred. A written follow up will be sent to the NIDA PO and NYU Grossman SoM IRB within 72 hours of the event. The written follow up will include information on the date of the event, what occurred, actions taken by project staff, planned follow up (if any), the intervention group of the affected participant, whether the event appears to be related to the intervention, and whether the participant will continue in the study. When additional clinical information becomes available, a follow-up and/or final SAE report will be filed with the IRB and NIDA.

Subjects may be withdrawn from the study if continued participation would be harmful or for non-compliance with study procedures or for a serious adverse reaction to study procedures. The study may be discontinued on the recommendation of the PI or the IRB.

4.4. Potential benefits to the subjects
There are no direct benefits to participants. Interview participants will receive modest incentive payments that compensate them for the time spent in study activities. The community at large, including patients and providers, benefits from knowledge gained about substance use treatment interventions and medical practice that is gathered through this study. The risks to subjects mentioned above are reasonable in relation to these anticipated benefits.

5. Subject identification, recruitment and consent

5.1. Method of subject identification and recruitment
A purposeful sampling approach will be used to select CATCH staff with a variety of roles, demographic characteristics, and backgrounds to participate in qualitative interviews. Staff will be recruited via meetings, email, and in-person, using an IRB-approved description of the study. Potential participants will be informed that interviews will be conducted outside of working hours in accordance with the participant's availability and preference.

Eligible patients will be identified from the EHR and/or by hospital staff (which could be members of the CATCH team) as hospitalized patients with a diagnosis of OUD. CATCH team members, other hospital staff, or research staff may offer patients a flyer notifying them of the opportunity to participate in an interview, and containing contact information for the research team. Research staff may speak with patients who have been identified by the CATCH team as potential interview candidates. Research staff will explain the study, offer participation, and obtain informed consent. Interviews will be conducted by research staff on hospital premises or at an off-site location following hospital discharge depending on patients' availability. During the COVID-19 pandemic, in-person patient interviews will occur in accordance with institutional policies (including travel restrictions, personal protective equipment/PPE and social distancing policies).

5.2. Process of consent
Potential participants will receive an IRB-approved Information Sheet which discloses to the potential participant the purpose of the study and the participant's rights of confidentiality. Potential participants will meet with research staff in a private room, where the contents of the information sheet will be reviewed in detail and the potential participant will be given an opportunity to ask any questions. Following this discussion, consent to participate in the study, including verbal consent for the interview and written consent for audio recording, will be obtained.

5.3. Subject capacity
Prior to enrollment, subjects will be asked to describe the activities required for study participation. Those who are unable to describe these study activities without guidance from the researchers will not be permitted to enroll. Study staff will err on the side of caution (i.e. lack of eligibility) in deciding if someone is cognitively able to give consent and participate in the study.

5.4. Consent forms and Documentation of Consent
Consent documents will be in English using an IRB-approved consent form that includes name and contact information of the PI and of the institutional review board, a description of the study, the payment schedule, a description of potential risks and benefits, a statement of confidentiality, an indication of the right to refuse or withdraw at any time without any consequence, and consent for audio recording. Staff consent forms will inform potential participants that a decision not to participate will have no bearing on the individual's employability, reputation, professional relationships, or relationship with NYU, New York City Health and Hospitals, or any affiliated individuals. Patient consent forms will inform individuals that their decision to participate will not affect the care they receive. Participants will be asked to provide verbal consent; a waiver of documentation of consent is requested, because the consent document would be the only record linking the participant to their participation in the research. Along with verbal consent, subjects will be offered the option of having their name recorded in writing as a study participant, but this is entirely voluntary. Any names are recorded in writing for this purpose will not be linked to a Study ID and will not appear on any study documents. Any names recorded, as well as signed consent forms for audio recording, will be filed in a secured cabinet located in the offices of the research team at NYU Grossman School of Medicine.

5.5. Costs to the subject
There are no costs to the subjects associated with this study.

5.6. Payment for participation
Incentives will be provided to participants in the form of cash or a gift card in the amount of $50. This payment amount is selected based on typical payment schedules for interviews with similar participant groups that were used by the PI in prior studies.

6. Research team

The study is led by PI Jennifer McNeely, MD, MS at the NYU Grossman SoM. Key personnel from outside NYU Grossman SoM are from the NYC Health and Hospitals (H+H) system, NYC Dept. of Health and Mental Hygiene (DOHMH), Weill Cornell Medicine (WCM), and Boston Medical Center (BMC).

NYU Grossman SoM study personnel will have research training including training in protection of human subjects, and will be directly supervised by the PI and Co-Investigators. All NYU Grossman SoM-affiliated study personnel have already taken or will take the mandatory Annual Regulatory training and Annual Compliance training for the NYU Grossman School of Medicine to ensure that they are aware of the importance of patient confidentiality and all appropriate laws regarding the protection against patient privacy breaches.

NYU:

Jennifer McNeely, MD, MS is Associate Professor in the Dept. of Population Health. Dr. McNeely's work focuses on the integration of substance use screening and interventions into general medical settings. She has conducted multiple studies in the H+H system, including NIDA-funded research for her K23 award, a NIDA Clinical Trials Network study, and a current R34.

Donna Shelley, MD, MPH (Co-I) is a national leader in health services research with particular expertise in implementation and dissemination science, and frequently collaborates with H+H and with Dr. McNeely.

Joshua Lee, MD, MS (Co-I), is a seasoned NIH researcher focused on studying the effectiveness of MAT in safety net patients, including criminal justice populations.

John Billings, JD, PhD (Co-I) is Professor of Health Policy and Public Service at the NYU Wagner School, and is an expert in the application of Medicaid claims and other administrative data sources to analyze patterns of hospital admission and emergency room visits in vulnerable populations, including individuals with substance use disorders.[16-18] He has studied characteristics of high cost Medicaid patients to inform the design of interventions to improve clinical care and outcomes.[19-22]

Thaddeus Tarpey, PhD, (Senior Biostatistician) is the PhD Program Director in the Division of Biostatistics in the Dept. of Population Health. He is an expert in developing precision medicine methodologies and works in designing innovative adaptive clinical trials.

Maria R. Khan, MPH, PhD is an Associate Professor in the Dept. of Population Health who has robust experience in using administrative data sources to understand social drivers of substance use and STI/HIV risk and outcomes in vulnerable populations.

Kumar Vasudevan, MD is a Research Fellow at NYU Grossman School of Medicine. His research involves developing and supporting novel ways to engage patients with substance use disorders. His work on the study is overseen by Drs. McNeely, Lee and Khan.

H+H:

Roopa Kalyanaraman Marcello, MPH, CPH (Co-I) is Asst. Vice President for Chronic Diseases and Prevention for the H+H system. Through her work in the OneCity Health unit of H+H, Ms. Marcello has extensive experience in the evaluation of clinical programs within the H+H system, and will oversee the extraction of data from the H+H clinical data warehouse for this study.

Charles Barron, MD (Co-I) is the former Deputy Chief Medical Officer –Behavioral Health for NYC Health + Hospitals, and acted as the H+H Lead Investigator for all six CATCH sites until the end of 2022. Dr. Barron previously worked as Interim Medical Director of Behavioral Health at NYC Health + Hospitals for 2 years, and in the Department of Psychiatry at Elmhurst Hospital Center for 25 years.

Other key contributors:

Ellenie Tuazon, MPH is a Senior Epidemiologist at the Bureau of Alcohol and Drug Use Prevention, Care and Treatment for the NYC DOHMH.

Gretchen Van Wye, PhD, MA is Assistant Commissioner & City Registrar, Bureau of Vital Statistics, NYC DOHMH.

Joe Kennedy, MPH is Director of the Data Matching Unit, Bureau of Vital Statistics, DOHMH

Zoe Weinstein, MD, MS at Boston Medical Center is a consultant who will provide technical assistance and advise on

the implementation of the CATCH intervention.
Ali Jalali, PhD is an Assistant Professor of Population Health Sciences, Weill Cornell Medicine. Dr. Jalali is a health economist and will contribute to the economic evaluation study of CATCH.
Sean M. Murphy, PhD is an Associate Professor of Population Health Sciences, Weill Cornell Medicine. He will also advise on the economic evaluation component of this study.

1. *US Dept. of Health and Human Services (HHS), Office of the Surgeon General, Facing Addiction in America: The Surgeon General's Report on Alcohol, Drugs, and Health*. 2016: Washington, DC.
2. Rudd, R.A., Seth, P., David, F., and Scholl, L. Increases in Drug and Opioid-Involved Overdose Deaths - United States, 2010-2015. MMWR Morb Mortal Wkly Rep. 2016:65(5051):1445-1452.
3. Ronan, M.V. and Herzig, S.J. Hospitalizations Related To Opioid Abuse/Dependence And Associated Serious Infections Increased Sharply, 2002-12. Health Aff (Millwood). 2016:35(5):832-7.
4. Park-Lee, E., Lipari, R.N., Hedden, S.L., Copello, E.A.P., and Kroutil, L.A., *Receipt of Services for Substance Use and Mental Health Issues among Adults: Results from the 2015 National Survey on Drug Use and Health*, in *NSDUH Data Review*. 2016.
5. Curran, G.M., Bauer, M., Mittman, B., Pyne, J.M., and Stetler, C. Effectiveness-implementation hybrid designs: combining elements of clinical effectiveness and implementation research to enhance public health impact. Med Care. 2012:50(3):217-26.
6. Glasgow, R.E., Lichtenstein, E., and Marcus, A.C. Why don't we see more translation of health promotion research to practice? Rethinking the efficacy-to-effectiveness transition. Am J Public Health. 2003:93(8):1261-7.
7. Hemming, K., Haines, T.P., Chilton, P.J., Girling, A.J., and Lilford, R.J. The stepped wedge cluster randomised trial: rationale, design, analysis, and reporting. BMJ. 2015:350:h391.
8. Brown, C.H., et al. An Overview of Research and Evaluation Designs for Dissemination and Implementation. Annu Rev Public Health. 2017:38:1-22.
9. Barker, D., McElduff, P., D'Este, C., and Campbell, M.J. Stepped wedge cluster randomised trials: a review of the statistical methodology used and available. BMC Med Res Methodol. 2016:16:69.
10. Glasgow, R.E., Vogt, T.M., Boles, S.M. . Evaluating the Public Health Impact of Health Promotion Interventions: The RE-AIM Framework. American Journal of Public Health. 1999:89(9):1322-1327.
11. Spear, S.E., Shedlin, M., Gilberti, B., Fiellin, M., and McNeely, J. Feasibility and acceptability of an audio computer-assisted self-interview version of the Alcohol, Smoking and Substance Involvement Screening Test (ASSIST) in primary care patients. Subst Abus. 2016:37(2):299-305.
12. McNeely, J., et al. Barriers and facilitators affecting the implementation of substance use screening in primary care clinics: a qualitative study of patients, providers, and staff. J Gen Intern Med. 2017:32(Suppl 2)(83):S128.
13. Kim, H.M., et al. Validation of key behaviourally based mental health diagnoses in administrative data: suicide attempt, alcohol abuse, illicit drug abuse and tobacco use. BMC Health Serv Res. 2012:12:18.
14. Steele, L.S., Glazier, R.H., Lin, E., and Evans, M. Using administrative data to measure ambulatory mental health service provision in primary care. Med Care. 2004:42(10):960-5.
15. Valenstein, M., et al. Targeting quality improvement activities for depression. Implications of using administrative data. J Fam Pract. 2000:49(8):721-8.
16. Raven, M.C., Carrier, E.R., Lee, J., Billings, J.C., Marr, M., and Gourevitch, M.N. Substance use treatment barriers for patients with frequent hospital admissions. J Subst Abuse Treat. 2010:38(1):22-30.
17. Raven, M.C., Billings, J.C., Goldfrank, L.R., Manheimer, E.D., and Gourevitch, M.N. Medicaid patients at high risk for frequent hospital admission: real-time identification and remediable risks. J Urban Health. 2009:86(2):230-41.
18. Billings, J. and Raven, M.C. Dispelling an urban legend: frequent emergency department users have substantial burden of disease. Health Aff (Millwood). 2013:32(12):2099-108.
19. Billings, J. Management matters: strengthening the research base to help improve performance of safety net providers. Health Care Manage Rev. 2003:28(4):323-34.
20. Billings, J., Dixon, J., Mijanovich, T., and Wennberg, D. Case finding for patients at risk of readmission to hospital: development of algorithm to identify high risk patients. Bmj. 2006:333(7563):327.
21. Billings, J., Georghiou, T., Blunt, I., and Bardsley, M. Choosing a model to predict hospital admission: an observational study of new variants of predictive models for case finding. BMJ Open. 2013:3(8):e003352.
22. Billings, J. and Mijanovich, T. Improving the management of care for high-cost Medicaid patients. Health Aff (Millwood). 2007:26(6):1643-54.
