# Supplementary material for: Addiction Consult Services, Mortality, and Acute Care Utilization in Inpatients With Opioid Use Disorder: A Secondary Analysis of a Cluster Randomized Clinical Trial
Source: JAMA Netw Open. 2025 Aug 6;8(8):e2525222. doi: 10.1001/jamanetworkopen.2025.25222 (PMC12329607; doi:10.1001/jamanetworkopen.2025.25222)
Supplement: Supplement 2. — eFigure 1. Timeline of study periods and outcome measures eFigure 2. Survival probability plots for all-cause, overdose, and opioid-involved overdose deaths [file jamanetwopen-e2525222-s002.pdf]

Supplementary Online Content

Rostam-Abadi Y, Wang S, King C, et al. Addiction consult services on 1-year mortality and acute care utilization in inpatients with opioid use disorder: a secondary analysis of a cluster randomized clinical trial. *JAMA Netw Open*. 2025;8(8):e2525222.
doi:10.1001/jamanetworkopen.2025.25222

eFigure 1. Timeline of study periods and outcome measures

eFigure 2. Survival probability plots for all-cause, overdose, and opioid-involved overdose deaths

This supplementary material has been provided by the authors to give readers additional information about their work.

© 2025 Rostam-Abadi Y et al. *JAMA Network Open*.

eFigure 1. Timeline of study periods and outcome measures

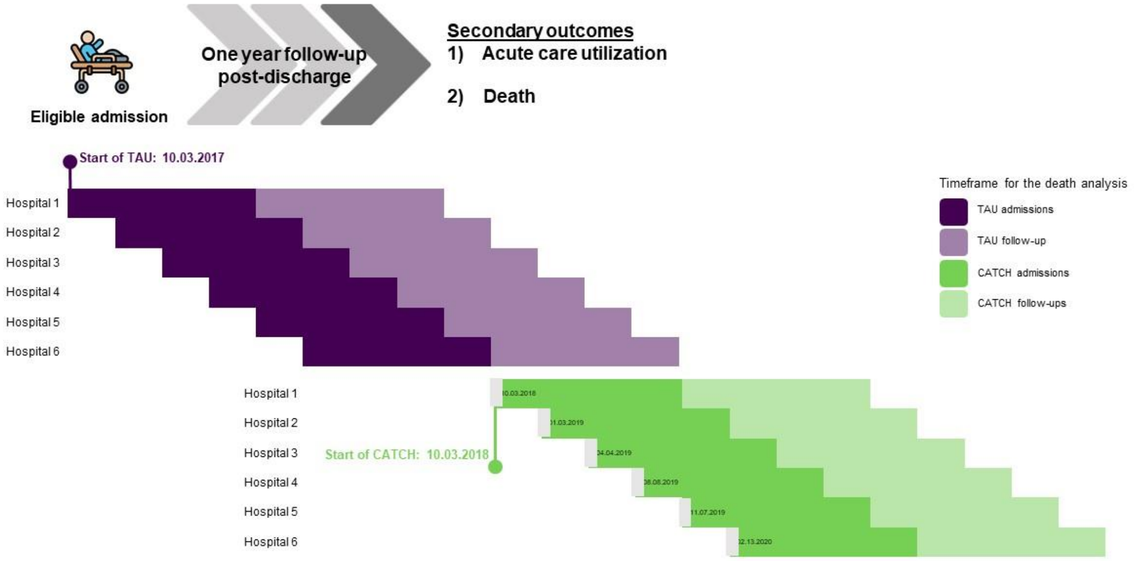

Notes
1. Dates represent the start date of the CATCH program at each hospital.
2. Gray boxes represent the 1-month transition period during which the CATCH program was introduced at each hospital.

© 2025 Rostam-Abadi Y et al. *JAMA Network Open*.

eFigure 2. Survival probability plots for all-cause, overdose, and opioid-involved overdose deaths

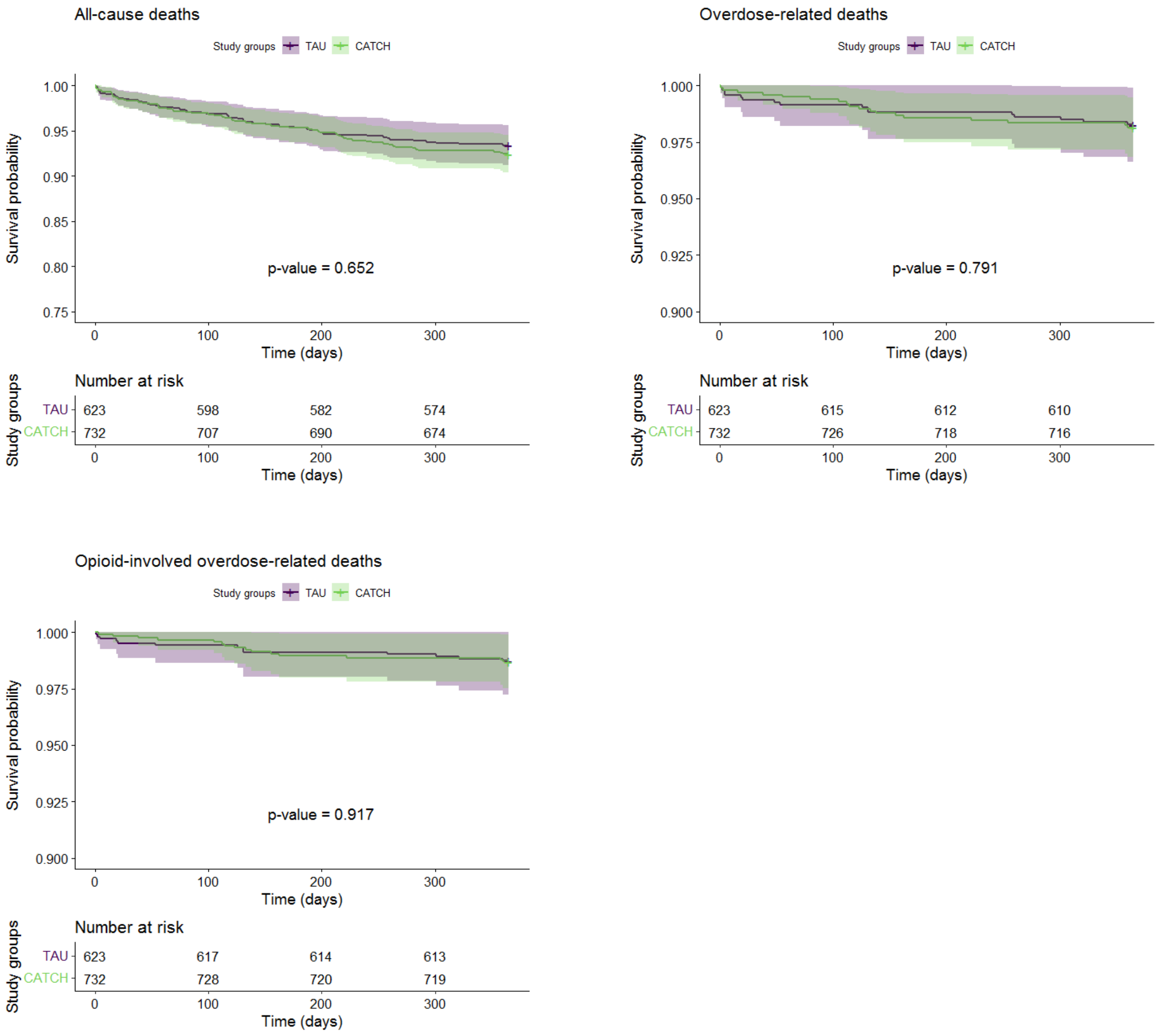

© 2025 Rostam-Abadi Y et al. *JAMA Network Open*.
