# Supplementary material for: Addiction Consult Services, Mortality, and Acute Care Utilization in Inpatients With Opioid Use Disorder: A Secondary Analysis of a Cluster Randomized Clinical Trial
Source: JAMA Netw Open. 2025 Aug 6;8(8):e2525222. doi: 10.1001/jamanetworkopen.2025.25222 (PMC12329607; doi:10.1001/jamanetworkopen.2025.25222)
Supplement: Supplement 3. — Data Sharing Statement [file jamanetwopen-e2525222-s003.pdf]

Data Sharing Statement

Rostam-Abadi. Addiction Consult Services on 1-Year Mortality and Acute Care Utilization in Inpatients With Opioid Use Disorder. *JAMA Netw Open*. Published August 06, 2025. doi:10.1001/jamanetworkopen.2025.25222

Data
Additional Information: ClinicalTrials.gov Identifier: NCT03611335
Data available: No

Additional Information
Explanation for why data not available: The main source of our study data is the New York State Medicaid claims. Due to their stringent confidentiality requirements, we are not able to make study data available.
